# Joint modelling of anaemia and stunting in children less than five years of age in Lesotho: a cross-sectional case study

**DOI:** 10.1186/s12889-022-12690-3

**Published:** 2022-02-11

**Authors:** Rugiranka Tony Gaston, Faustin Habyarimana, Shaun Ramroop

**Affiliations:** 1 School of Mathematics, Statistics and Computer Sciences, University of KwaZulu-Natal, Private Bag X01, Pietermaritzburg CampusScottsville, 3209 South Africa; 2 Health Economics and HIV/AIDS Research Division (HEARD), University of KwaZulu-Natal, Westville Campus, Private Bag X01, Westville, 3629 South Africa

**Keywords:** Malnutrition, Anaemia, Correlation, Multivariate joint model, Children less than 5 years of age and LDHS

## Abstract

**Background:**

Anaemia and stunting remain jointly a serious health issue worldwide especially in developing countries. In Lesotho, their prevalence is high, particularly among children less than 5 years of age.

**Objectives:**

The primary objective was to determine the association between anaemia and stunting, and identify factors relating to both conditions among children younger than 5 years in Lesotho.

**Methods:**

This cross-sectional study used secondary data from 3112 children collected during the 2014 Lesotho Demographic Health Survey (LDHS). Haemoglobin (Hb) levels were adjusted for altitude and a level less than 11 g per deciliters (11 g/dl) was determined as the cutoff for being anaemic. A child with the height-for-age z score (HAZ) below minus two standard deviations (SD) was considered to have stunting. We linked factors relating to anaemia and stunting using a multivariate joint model under the scope of the generalized linear mixed model (GLMM).

**Results:**

The prevalence of anaemia and stunting in children younger than 5 years were 51% and 43% respectively. The multivariate results revealed a strong association between anaemia and stunting. In addition, maternal education, urban vs. rural residence, wealth index and childbirth weight significantly impacted childhood stunting or malnutrition, while having fever and/or diarrhoea was linked to anaemia. Lastly, age was shown to have a significant effect on both stunting and anaemia.

**Conclusion:**

Anaemia and stunting or malnutrition showed linked longitudinal trajectories, suggesting both conditions could lead to synergetic improvements in overall child health. Demographic, socio-economic, and geographical characteristics were also important drivers of stunting and anaemia in children younger than 5 years. Thus, children living in similar resources settings as Lesotho could benefit from coordinated programs designed to address both malnutrition and anaemia.

## Introduction

Malnutrition and anaemia remain major health problems worldwide especially in developing countries [5, 26, 48]. These conditions intersect and are linked to morbidity and mortality worldwide, particularly in pregnant women and children [6, 10, 22]. Despite progress in the fight against childhood malnutrition and anaemia, several challenges remain. Globally, in 2017, 151 million children younger than 5 years. Among those children, three quarters were from the South-East Asian and African regions [42]. In 2011, the global prevalence of anaemia in children younger than 5 years was approximately 43%, with highest percentages also found in the African and South Asian regions [24, 30, 47].

In Lesotho, childhood anaemia and malnutrition remain a concerning health problem with 51% of children being anaemic and 53% malnourished [31]. Improving both the nutritional and anaemic status in children younger than 5 years is critical to ensure high quality of life to future contributors and leaders of the country [14].

Anaemia is defined by low blood level of haemoglobin (Hb). According to the World Health Organization (WHO), children younger than 5 years are considered anaemic when their Hb level, adjusted for altitude, is less than 11 g/dl [17, 24], WHO 2015. The cause of anaemia is multifactorial and iron deficiency is considered the most fundamental cause in about 50% of cases. Insufficient folate, vitamin B12, protein deficiencies, nutrients and some diseases, such as malaria and diarrhoea among others, can also increase the risk of anaemia [12, 14]. Childhood anaemia can negatively impact cognitive development, performance in school, physical growth and immunity [14, 25, 40].

Malnutrition develops with either over- or under consumption of food but herein, it is defined as an insufficient intake of nutrients and/ or other minerals. In developing countries, a low nutritional status of a child is an indicator of health problems [29]. Childhood malnutrition leads to long-term negative effects, such as poor performance at school, delayed psychomotor development, lower capacity for work and reduced quality of life in adulthood [10, 21, 34]. The nutritional status of a child is mainly evaluated based on different anthropometric indicators in reference to WHO growth standards [31]. Stunting (height-for-age) indicates chronic or long-term malnutrition, wasting (low-weight-for-height) is linked to low food intake and/or illness and is described as acute malnutrition, while an underweight child (weight-for-age) can be either stunted, wasted or both [31]. The study examines the association between anaemia and stunting as an indicator of long-term malnutrition [3].

According to the WHO, an individual whose z-score falls below -2 SD is considered malnourished [46]. Stunting is categorised as moderate acute malnutrition (MAM) when the height-for-age z score (HAZ) is less than minus two SD, and severe acute malnutrition (SAM) when HAZ is below minus two SD [7, 8, 23, 31]. Literature indicates that most studies have evaluated anaemia and stunting independently [14, 28, 38, 45]. The independent models are sufficient in modelling anaemia and stunting but are inadequate for addressing the association between the two conditions. The generalized linear mixed model (GLMM) were extended to evaluate joint trajectories of repeated measures [11, 33],where the random effect can be used to evaluate the correlation structure between several response variables and can better control for type 1 error [4, 15, 20]. In addition, the multivariate joint model has the ability to address multivariate questions [18, 33].

The studies aimed to understand the association between anaemia and stunting in children younger than 5 years are limited [2, 32, 36, 37, 39, 43, 48]. We found no study in literature that has utilized the joint model for anaemia and stunting in children younger than 5 years of age in Lesotho. Therefore, we expanded existing models by examining the longitudinal interdependent relationship of anaemia and stunting among children younger than 5 years in Lesotho using joint multivariate GLMM. Understanding the link between anaemia and stunting and other factors will help prioritize efforts for policy-makers and donors that aim to improve global child health.

## Materials and methods

### Study area

Lesotho is small landlocked country with a surface area of 30355km^2^ and a population of about of 2.2 million and is surrounded by only South Africa. Lesotho has ten politico-administrative districts with Maseru as the capital city, and is ruled by a King as the head of state and the Prime Minister as head of the government. The kingdom of Lesotho is known for its abundant water resources and high altitude. However, the country has high unemployment rates, high prevalence of HIV and AIDS, poverty, food insecurity, and the burden from other diseases [31]. The country is also vulnerable to natural disasters and climate change such as droughts and heavy rain and flooding (Renzaho 2015).

### Data source and sampling techniques

This study was cross-sectional and used secondary data from the LDHS, conducted from September to December 2014. The ethical approval for the 2014 LDHS was assessed and confirmed by the Lesotho Ministry of Health Research and Ethics Committee together with support of the Institutional Review Board of ICF International.

The 2014 LDHS was representative at national, urban and rural areas as well as four ecological zones, and each of Lesotho’s 10 districts [31]. The sample was stratified and selected in two stages. The stratification was executed by separating each district into urban and rural areas. The overall of 20 sampling strata were designed, and thereafter samples were selected independently in each sampling stratum by following a two-stage sampling method. The first stage included a random selection of 400 clusters from the enumeration area (EAs). Out of the 400 clusters, 118 clusters were from urban areas and 282 clusters from rural areas. The second stage involved systematic sampling of 9942 randomly selected households covering all EAs. Out of these, 25 households were then selected from each enumeration area [31].

The 2014 survey included all residents or visitors who were in the selected household the night before the interview. All women were included in the survey based on the condition that they had never been married, not currently pregnant, and had not given birth in the previous 2 months. Children aged between 0–59 months from mothers living at or visiting the households the night before were included in survey. All surveyed children were measured for height and tested for anaemia, under the supervision of their parents or guardians. Exclusion criteria, comprised the anomaly in BMI, height, and weight-for-age measurements. With respect to anaemia testing, all children less than 6 months were not included in the survey. The present study used a total weighted number of 1138 children for anaemia, and 1297 for stunting. The exclusion and system missing values were considered as missing values and were consequently ignored [31].

The calculation of sample size was determined using the statistical formula:

$$n=\frac{{z}^{2}p\left(1-p\right)}{{d}^{2}}$$

where *n* = sample size, *p* = prevalence of anaemia, z = z-value at 90% confidence (= 1.96), and d = level of significance (= 5%). Out of the total weighted of 3112 children drawn as a sample, only 1292 children were used in this study, and 1816 were considered as the missing value.

In the sampling procedure, the number of women surveyed in each region should present the size of the total sample in the proportion to the size of the region [31]. However, some regions such as Qacha’s Nek, Quthing are less populated, while Maseru and Leribe are heavily populated. Since the population in each region was not of equally weighting, individuals surveyed in each region should contribute equivalently to the total per region. Hence, this unweighted distribution does not accurately represent the exact population. Therefore, the weighted samples were used to infer the national status and account for the complex sample design from the LDHS data set as well as to account for the lack of adequate representation in the sample. More details on calculation of sampling size and sampling weight can be found in Lesotho’s report of demographic and health survey [31].

### Anaemia and nutrition assessment

#### Anaemia testing

All children younger than 5 years surveyed were tested for anaemia under the supervision of their parents or guardians. Blood collection by finger-or heel-prick was performed by professional nurses using a spring-loaded sterile lancet. A blood drop was gathered in microcuvette, and the Hb was measured using a HemoCue analyser (company, city of equipment). The lancet, microcuvette, gloves and alcohol swabs were used once for hygiene safety. Results were obtained within 10 min and shared verbally with the children’s parents or guardians and recorded as a hard copy, captured on the Biomarker questionnaire and indicated on handout that explains causes and counteractions of anaemia, which were left with the family [31].

All parents or guardians of children with a Hb level less than 7 g/dl were told to take the children to the nearest healthcare facility for follow-up [31].

#### Nutrition status assessment

Height was measured with a tape board and weight with an electronic balance (model of equipment, city, country) provided by UNICEF. The weight of the children was estimated utilizing a Seca gauging scale (model of equipment, city, country), which aligned to zero. For weighting, parents or guardians unclothed their children or keep them in light clothing. For children unable to stand, child weight was calculated based on difference in parent weight compared to weight of parent holding child. The height measurement was carried out using a short board, which was lying down or standing on a level ground surface. Children were measured without shoes and headgear, standing against a board. Children less than 87 cm were measured in supine position. From the children’s weight, height and age was calculated their nutritional status (i.e. weight-for-age, height-for-age, and weight-for-height) based on the WHO guidelines [9, 12, 31].

## Data analysis

### Dependent variable

The two response variables for the study were measures of anaemia and stunting. Anaemia in children can be grouped as severe anaemia when Hb level is less than 7 g/dl; moderate anaemia with Hb level between 7 and 8.9 g/dl; and mild anaemia with Hb level between 9 and 10.9 g/dl. However, in the scope of this study, a child was determined as either as anaemic or not (i.e., Hb level above or below altitude adjusted threshold of 11 g/dl) (WHO 2015). Nutrition status of a child cab be described as non-malnourished; moderate acute malnutrition; or severe acute malnutrition. Yet, the second response variable was recorded as binary exposure since the interest of the study was to check if the child is stunted or normal [23, 31].

### Independent variables

The independent variables include socio-economic and demographic factors, and were used in previous studies on childhood anaemia or stunting [14, 15, 28, 39]. Demographic variables included sex of child (male or female),whether the child had fever, cough or diarrhoea in the 2 weeks prior to the survey; the birth order of the child; the childbirth weight; and the age in months. Socio-economic variables included reception of Vitamin A supplementation in the 6 month prior to the survey; whether the child visited a health facility in past 2 weeks prior to the survey; maternal education (no education, primary, post primary); place of residence (urban, rural); whether a household used electricity; main material of floor, wall, and roof; wealth index of the household; mother’s body mass index (BMI); toilet facility; and source of drinking water.

## Statistical analysis

Multivariate joint GLMM models were built to identify the correlation between two response variables (anaemia and stunting) and assess their association with demographic and socio-economic factors. Using Statistical Package for the Social Sciences (SPSS version 25.0), we cross tabulated and summarised the data with frequencies and percentages. We then conducted univariate analysis to selected variables showing relationship to either responses variables based having a *p*-value less than 0.2; this selection helps to account for multicollinearity and confounders between covariates [12–14].

Selected variables were then included in the multivariate joint GLMM conducted with SAS 9.4 using PROC GLIMMIX. This procedure is able to join models with two response variables that have similar distribution and link function. Based on the convergence criteria, the unstructured (UN) convergence was chosen seemed to be the best for the analysis. The final multivariate joint analysis retained independent variables showing *p*-value < 0.05. Interactions were evaluated based on Akaike information criteria (AIC); but were not significant [15, 33].

### Model formulation

Two responses variables, stunting and anaemia in children less than 5 years of age were examined: the first variable ${x}_{i1}$ was stunting, where one (1) indicate the presence stunting and zero its’ absence; and the second ${x}_{i2}$ allocate a one to the presence of anaemia, versus zero (0) for its’ absence. We assume the outcome to be from a bivariate Bernoulli distribution, with ${p}_{i1}$ as the likelihood of stunting occurring in a child *i* and ${p}_{i2}$ as the probability of anaemia occurring in a child *i.* Therefore, the binary generalized linear model can be expressed as follow:

1 $${f}_{1}\left({\psi }_{i1}\right)={Y}_{i1}{\theta }_{1}+{Z}_{i1}{u}_{1}$$

2 $${f}_{2}\left({\psi }_{i1}\right)={Y}_{i2}{\theta }_{2}+{Z}_{i2} {u}_{2}$$

where, ${\theta }_{1}$ and ${\theta }_{2}$ are assumed to be the vectors of fixed effects, ${u}_{1}$ and ${u}_{2}$ are the vectors of the random effects. In addition,${Y}_{i1}$,${Y}_{i2}{, Z}_{i1}$ and ${Z}_{i2}$ are the designed matrices for fixed and random effects, respectively. Hence, the model’s equation of the variance–covariance matrices for multivariate normal distribution (MVN) is shown as follows:

3 $$U=\left(\begin{array}{c}{u}_{1}\\ {u}_{2}\end{array}\right)\sim i.i.d.MVN \left(0,\sigma \right)=MVN\left(\left[\begin{array}{c}0\\ 0\end{array}\right],\left[\begin{array}{cc}{\sigma }_{11}& {\sigma }_{12}\\ {\sigma }_{21}& {\sigma }_{22}\end{array}\right]\right)$$

where the ${\sigma }_{11}$ and ${\sigma }_{22}$, are the variance components of stunting and anaemia respectively, while ${\sigma }_{12}$ and ${\sigma }_{21}$ are the covariance components between these conditions. When the covariance components from Eq. (3), ${\sigma }_{12}$= ${\sigma }_{21}$=0, the multivariate joint model becomes standard separated generalized linear mixed [19, 33].

### Model testing and and goodness-of-fit

#### Covariance structure of the random effects

The test of random effect checks whether specific covariates should be included within the random effect structure of a model by testing the model against the hypothesis that the variance of population distribution is zero. To illustrate, assume that we want to test the variance parameter ${\sigma }^{2}$ for a GLMM with a single random effect: Since the variance cannot be negative, zero is the boundary of the parameter space, and model variance can be tested against zero using a one‐sided hypothesis [41, 44].

The null hypothesis to be tested is ${H}_{0}:{\sigma }^{2}=0$, against ${H}_{1}:{\sigma }^{2}\ne 0$, and this can tested using the likelihood ratio test, the Wald test or the score test. These are asymptotically comparable under the null hypothesis and follow a distribution with a given degree of freedom. In addition, these tests can determine if the random effects in a GLMM contribute [41, 44].

#### Goodness‐of‐fit tests

The goodness-of-fit can be evaluated by a likelihood ratio test against an alternative saturated model, which is the most complex model explained by the data. The saturated model can be expressed as ${{\varvec{E}}({\varvec{Y}}}_{{\varvec{n}}{\varvec{i}}}/{\varvec{\beta}},{{\varvec{\theta}}}_{{\varvec{n}}})={{\varvec{Y}}}_{{\varvec{n}}{\varvec{i}}}$ for all *n* and *i.* When the dependent variables are categorical, the goodness-of-fit test for the data is Pearson chi-squared test [41]. The Pearson chi-squared test can be written as follows:

4 $${\upchi }^{2}=\sum_{i=1}^{I}{n}_{i}\sum_{j=0}^{J}\frac{{\left({y}_{ij}-{\widehat{\pi }}_{ij}\right)}^{2}}{{\widehat{\pi }}_{ij}}$$

where ${n}_{i}$ is the number of participants with the same values on the covariates, ${y}_{ij}$ and ${\widehat{\pi }}_{ij}$ are respectively the observed and expected quantity of the participants in the group *i* responding in category *j* [41].

## Results and interpretation

### Univariate results

This study included a total weighted number of 3112 children aged between 0–59 months. The prevalence of anaemia and stunting was 51% and 43% respectively, with 35.2% of children having both conditions. Tables 1 and 2 indicate the frequency and percentage of anaemia with corresponding independent variables. Childhood stunting and anaemia were both related to child age but in opposing direction (i.e., stunting increased with age, while anaemia decreased with age), while stunting itself was further associated with sex, having visited healthcare facilities, maternal education, wealth index, access to electricity, drinking water and waste water management, and dwelling characteristics (wall and floor material); while anaemia was more specifically related to fever in last 2 weeks, recent diarrhoea, and roof characteristics of the dwelling. The prevalence of anaemia was higher in children aged between 0–19 months (62.7%), and then decreased in children aged 20–39 months (55.0%) and then again in those aged 40–59 months (44.4%). The anaemia prevalence was higher in children who experienced fever in the last 2 weeks (62.8%) compared to children who did not (37.2%). The prevalence of stunting was lower in younger children (age group 0–19 months, 17.8%), The prevalence of stunting was higher from mothers with low education (72.7%), but increased as children aged (20–39 months, 33.3%; and 40–59 months, 31.2%).

**Table 1:** Childhood anaemia by categorical variable

Variables	Categories	Anaemic	Not anaemic	*p*-value
Sex of the child	Male Female	56.2% (301) 52.2% (315)	43.8% (235) 47.8% (288)	0.185
Child’s age in months	6–19 20–39 40–59	62.7% (227) 55.0% (224) 44.4% (164)	33.3% (135) 45.0% (183) 55.6% (205)	0.000
Child’s birth weight	< 2500 g ≥ 2500 g	58.5% (48) 53.2% (467)	41.5% (34) 46.8% (410)	0.359
Had fever in last 2 weeks	Yes No	62.8% (120) 52.3% (493)	37.2% (71) 47.7% (449)	0.008
Had diarrhoea recently	Yes No	62.7% (94) 52.8% (520)	37.3% (54) 47.2% (465)	0.024
Had cough in last 2 weeks	Yes No	54.4% (184) 54.0% (429)	45.6% (154) 46.0% (366)	0.883
Received vitamin A in last 6 months	Yes No	54.3% (357) 53.7% (253)	45.7% (301) 46.3% (218)	0.858
Birth order	1^st^ 2–3 4–5 ≥ 6	53.2% (231) 56.0% (261) 52.1% (85) 51.3% (39)	46.8% (203) 44.0% (205) 47.9% (78) 48.7% (37)	0.728
Mother’s BMI	< 18.5 ≥ 18.5	56.0% (14) 53.8% (596)	44.0% (11) 46.2% (511)	0.830
Mother’s education level	No education Primary Post primary	53.8% (7) 51.1% (267) 56.6% (341)	46.2% (6) 48.9% (255) 43.4% (262)	0.193
Visited health facility	Yes No	52.7% (479) 59.4% (136)	47.3% (430) 40.6% (93)	0.069
Wealth Index	Poor Middle Rich	58.0% (280) 53.1% (127) 50.1% (209)	42.0% (203) 46.9% (112) 49.9% (208)	0.059
Place of residence	Rural Urban	55.5% (457) 50.2% (158)	44.5% (366) 49.8% (157)	0.104
Household with electricity	Yes No	50.0% (150) 55.5% (465)	50.0% (150) 44.5% (373)	0.102
Toilet facility	Toilet with flush Pit latrine No facility	53.7% (306) 48.3% (87) 57.1% (222)	46.3% (264) 52.7% (93) 42.9% (167)	0.148
Type of drinking water	Tap water Protected water Unprotected	54.0% (274) 49.1% (109) 57.0% (233)	46.0% (233) 50.9% (113) 43.0% (176)	0.166
Main roof material	Thatch/Palm leaf Corrugated metal Stick &mud	58.8% (248) 52.3% (335) 42.7% (32)	41.2% (174) 47.7% (306) 57.3% (43)	0.014
Main wall material	Wood/Mud Bricks Cement /Block	56.6% (233) 51.5% (202) 52.6% (120)	43.4% (179) 48.5% (190) 47.4% (108)	0.270
Main floor material	Earth/Sand Mud block/Wood Cement/ Block	52.8% (152) 56.6% (233) 52.5% (229)	47.2% (136) 43.4% (179) 47.5% (207)	0.441

**Table 2:** Childhood stunting by categorical variable

Variables	Categories	Stunted (Malnourished)	No stunted (Nourished)	*p*-value
Sex of the child	Male Female	30.7% (184) 23.0% (160)	69.3% (416) 77.0% (537)	0.002
Child’s age in months	0–19 20–39 40–59	17.8% (92) 33.3% (136) 31.2% (115)	82.2% (426) 66.7% (273) 68.8% (254)	0.000
Child’s birth weight	< 2500 g ≥ 2500 g	50.6% (45) 22.4% (227)	49.4% (44) 78.6% (787)	0.000
Had fever in last 2 weeks	Yes No	21.2% (44) 27.6% (299)	78.8% (164) 72.4% (785)	0.054
Had diarrhoea recently	Yes No	27.2% (44) 26.5% (299)	72.8% (118) 73.5% (830)	0.855
Had cough in last 2 weeks	Yes No	26.1% (97) 26.7% (246)	73.9% (275) 73.3% (674)	0.807
Received vitamin A in last 6 months	Yes No	26.0% (176) 27.2% (166)	74.0% (500) 72.8% (444)	0.633
Birth order	1^st^ 2–3 4–5 ≥ 6	24.5% (126) 27.0% (145) 29.8% (51) 28.4% (21)	75.5% (388) 73.0%(392) 70.2%(120) 71.6% (53)	0.529
Mother’s BMI	< 18.5 ≥ 18.5	29.6% (8) 26.3% (333)	70.4% (19) 73.7% (931)	0.702
Mother’s education level	No education Primary Post primary	72.7% (8) 31.3% (186) 21.7% (150)	27.3% (3) 68.7% (408) 78.3% (542)	0.000
Visited health facility	Yes No	24.4% (257) 35.7% (87)	75.6% (796) 64.3% (157)	0.000
Wealth Index	Poor Middle Rich	33.8% (184) 30.1% (80) 16.3% (79)	66.2%(361) 69.9%(186) 83.7% (406)	0.000
Place of residence	Rural Urban	27.3% (257) 24.2% (86)	72.7% (683) 75.8% (270)	0.246
Household with electricity	Yes No	18.2% (64) 29.6% (279)	81.8% (288) 70.4% (665)	0.000
Toilet facility	Toilet with flush Pit latrine No facility	26.4% (170) 19.8% (41) 29.8%(133)	73.6% (473) 80.2% (166) 70.2% (133)	0.027
Type of drinking water	Tap water Protected water Unprotected	29.9% (173) 19.2% (48) 26.1% (122)	70.1% (406) 80.2% (202) 73.9% (345)	0.006
Main roof material	Thatch/Palm leaf Corrugated metal Stick & mud	34.4% (165) 23.2% (169) 11.1% (10)	65.6% (314) 76.8% (558) 88.9% (80)	0.000
Main wall material	Wood/Mud Bricks Cement /Block	33.0% (194) 20.1% (89) 22.6% (60)	67.0% (394) 79.9% (354) 77.4% (205)	0.000
Main floor material	Earth/Sand Mud block/Wood Cement/ Block	20.9% (72) 33.8% (158) 23.5% (114)	79.1% (272) 66.2% (309) 76.5% (372)	0.000

The prevalence of stunting was higher in children from mothers with low education (72.7%), and reduced as the level of mother’s education increased by primary (31.3%) and post-primary (21.7%) respectively.

### Multivariate results

Table 3 presents the estimation results for the fixed effects of the joint GLMM several socio-economic and demographics factors showed significant relationships with both stunting and anaemia. Child’s age had a significant effect on both anaemia and stunting. Children aged less than 20 months were less likely to be stunted (OR: 0.44, 95% CI: 0.298; 0.638) but did not differ in their risk of anaemia compared to the reference group (40–59 months). However, children aged 20–39 months did not differ in their risk of stunting but were more likely to be anaemic (OR: 1.7, 95% CI: 1.207; 2.396) compared to the reference. Fever and diarrhoea were not linked to stunting but were both associated with a higher risk of anaemia (OR: 0.491, 95% CI: 0.341; 0.707 and OR: 0.609, 95% CI: 0.410; 0.905, respectively). High birth weight (≥ 2500 g) and living in a rural area were protective against stunting (OR: 0.24, 95% CI: 0.182; 0.452 and OR: 0.52, 95% CI: 0.333; 0.814, respectively) compared to lower birthweight children; while the odds of being stunted increased with higher levels of poverty (OR: 3.5, 95% CI: 2.149; 5.703) compared to those from wealthier homes. Children from the middle wealth tertile were 2.9 times more likely to be stunted compared to those from the top wealth tertile. Lastly, lower maternal education was also associated with stunting.

**Table 3:** Parameter estimates for a joint marginal model for stunting and anaemia

Indicator variables	Stunting				Anaemia			
	**Estimate; SE**	**OR**	**95% CI**	***P*-value**	**Estimate; SE**	**OR**	**95% CI**	***P*-value**
**Child’s age**								
Ref: > 39 months	-	-	**-**	**-**	**-**	**-**	**-**	**-**
20–39 months	0.226;0.186	1.254	0.870;1.806	0.225	0.531;0.175	1.701	1.207;2.396	0.003
< 20 months	0.829;0.194	0.436	0.298;0.638	< 0.001	0.096;0.167	0.908	0.655;1.260	0.565
**Child had fever**								
Ref: Yes	-	**-**	**-**	**-**	**-**	**-**	**-**	**-**
No	0.153;0.217	1.165	0.762;1.782	0.482	-0.712;0.186	0.491	0.341;0.707	< 0.001
**Child had Diarrhoea**								
Ref:Yes	**-**	**-**	**-**	**-**	**-**	**-**	**-**	**-**
No	-0.227; 0.231	0.797	0.507;1.254	0.326	-0.496; 0.202	0.609	0.410;0.905	0.014
**Child’s birth weight**								
< 2500 g	-	-	-	**-**	**-**	**-**	**-**	**-**
≥ 2500 g	-1.248; 0.232	0.240	0.182;0.452	< 0.001	0.038; 0.230	1.039	0.662;1.631	0.868
**Residence**								
Ref: Urban	**-**	**-**	**-**	**-**	**-**	**-**	**-**	**-**
Rural	-0.653; 0.228	0.520	0.333;0.814	0.004	0.102; 0.197	1.107	0.753;1.629	0.603
**Wealth Index**								
Ref: Richer	**-**	**-**	-	**-**	**-**	**-**	**-**	**-**
Middle	1.065; 0.247	2.901	1.788;4.707	< 0.001	0.224; 0.199	1.251	0.847;1.848	0.262
Poorer	1.253; 0.249	3.501	2.149;5.703	< 0.001	0.282; 0.200	1.326	0.896;1.962	0.158
**Education level**								
Ref:No education	**-**	**-**	**-**	**-**	**-**	**-**	**-**	**-**
Primary	-1.473; 0.783	0.229	0.049;1.064	0.060	-0.772; 0.812	0.462	0.094;2.270	0.342
Post Primary	-1.841; 0.787	0.159	0.121;2.933	0.020	-0.519; 0.814	0.595	0.121;2.933	0.524

The variance components and covariance between anaemia and stunting are presented in Table 4. The covariance coefficient estimate of 1.000 indicated a positive relationship between the two conditions, meaning that changes in either nutrition or anaemia in a child impacts the likelihood of both diseases. In addition, the odds ratio of 2.718 confirmed that the two conditions are highly associated. The overall fitted model was highly significant as the coefficient of covariance parameter indicated the *p*-value < 0.001. Hence, including the random effect in the model was shown to be very important [16, 33], Zhang and Lin 2008.

**Table 4:** Variance Components and covariance between anaemia and stunting

Variables	Estimate; SE	OR	95% CI	*P*-value
Variance (stunting)	0.104; 0.010	1.110	1.088; 1.132	0.149
Variance (anaemia)	0.314; 0.170	1.369	0.981; 1.910	0.033
Covariance between anaemia and stunting	1.000; 0.141	2.718	2.063; 3.582	0.001

The test covariance parameters based on pseudo-likelihood rejected the null hypothesis of zero correlation with Pearson chi-squared test = 2644.470 and *p*-value < 0.001. This revealed that the association between anaemia and stunting was significant and not zero [41]. Furthermore, the results from the fitted statistics for conditional distribution indicated the Pearson chi-squared = 2123.070 with 0.94 ° of freedom. This is an indication of a good variability in the dataset and residual over dispersion was not present [16, 33].

## Discussion

This cross-sectional study used secondary data from 2014 LDHS. To our knowledge, this was the first study to simultaneously model the association between anaemia and stunting in children less than 5 years of age in Lesotho. The study utilized a multivariate joint model under the scope of GLMM to association both diseases and explore their associated socio-economic and demographic factors. Anaemia and stunting show a significant positive association confirming that the two diseases should be considered interrelated health problems in children where these diseases are more likely to coexist and interinfluence their manifestations. Thus, coordinated interventions aiming to improve both stunting and anaemia are likely to produce synergetic effects on child health. The association between the two conditions can be interpreted as an indication of chronic malnutrition which might cause iron deficiency. Similar results were described in studies by Yang et al. [48], Gari et al. [13], Mohammed et al. [32], Rahman et al. [37] and Rivadeneira et al. [39]. Our findings also indicate that child age has a significant effect on both anaemia and malnutrition but impact different age groups. The chance of having anaemia or stunting reduced as the children aged.

A possible explanation to this issue is due to the fact that the immune systems of children are still developing and consequently weak, hence need more nutrients to support the rapid body growth. In addition, many children at an early age are not breastfeed which makes them susceptible to various illness. Some of these conditions reduce the haemoglobin level within the blood which may lead to anaemia and stunting. Furthermore, as children age and are introduced to foods, and are able to consume a variety of nutrition, this would aid in putting them at less risk of being anaemic or stunting. Similar results we found in previous studies [3, 7, 13, 14, 24, 37]. However, the studies by Anticona and Sebastian [7] and Oliveira et al. [35] showed that stunting increased as the children grew older.

The findings from this study revealed that risk of anaemia was related to having experienced recent fever and diarrhoea. This may be due to the fact that fever and diarrhoea are commonly accompanied by the number of diseases and morbidity which are associated with anaemia. This has also been previously described [14, 19, 39]. The probability of being stunted reduced with increasing level of maternal education. The aforementioned could be linked to socio-economic status, where educated individuals are more likely to have a better standard of living, and knowledge of balanced diet. In addition, educated individuals can easily access and improve the nutritional status as most of them have a monthly income. This is also consistent with previous studies such as Kavosi et al. [22], Aheto et al. [5], Adebayo et al. [1], Aheto et al. [6], and Adhikari et al. [3].

Childbirth weight significantly impacts stunting in children, with lower risk in children born with a higher weight (≥ 2500 g). This relationship can be explained by the fact that children with low birth weight are more likely to have other co-morbidity illnesses that might be associated with stunting. Similar findings were found in studies by Yang et al. [48], Habyarimana et al. [19], and Kejo et al. [24].

We found that children living in rural areas have a lower risk of stunting, an effect that is debated in the field. A possible explanation to this is due to the fact that some individuals in rural areas are educated and they eat fresh food and fruits with more nutrients. Also, individuals from rural areas are breastfeed for a long period of time, which can contribute to fighting stunting at an early age. Some studies have described similar results, such as the study by Kavosi et al. [22], while others have described contrasting results Yang et al. [48] and El Kishawi et al. [12].

Lastly, children living within families from the middle and top tertile wealth index have a lower risk of having malnutrition. This further undergirds the fact that malnutrition is linked to socio-economic conditions, where the children from the lower wealth index cannot afford proper food, maintain hygiene and access to health care services. Similar results were found in previous studies such as the study by Gari et al. [13], Mohammed et al. [32], and Rivadeneira et al. [39].

### Strengths and limitation of the study

We used joint modelling to assess the association between anaemia and stunting in children less than 5 years of age. However, the present study has some limitation, the first being that the dataset was cross-sectional and it would be good to assess changes in disease trajectories and associated over time. The study used stunting variable as a longer term indicator of malnutrition [37] but other variables that reflect nutritional status could have also been assessed. Also, it would be interesting to use spatial joint models to assess the association between stunting and anaemia by geographical location. In addition, the food records would be mentioned in the study. Lastly, the study did not include the maternal anaemia levels, which are usually the biggest predictor of child anaemia. Hence, all the areas of focus not covered in this study, especially those mentioned, are considered limitations that will be addressed in future studies.

## Conclusion

This study aimed to determine the association between anaemia and stunting in children less than 5 years of age in Lesotho using multivariate joint model under GLMM. The study also assessed the association of socio-economic and demographic factors with anaemia and stunting. Lastly, we evaluated possible interaction effect between independent variables but none passed significance threshold. We found a significant positive association between anaemia and stunting which indicates that, when malnutrition increases in children less than 5 years, anaemia also increases and vice-versa. Thus, a change in childhood stunting can have a significant impact on anaemia status. In addition, several socio-economic and demographic factors impact both malnutrition and anaemia such as family wealth, maternal education, urban vs. rural living environments. In addition, children that were low birthweight or who have recently experienced fever, or diarrhoea should be prioritized for intervention. Knowledge on the relationship between anaemia and malnutrition together with other determinants can provide useful insights to policy makers, donors and government in planning and fighting to improve child through tailored public health messages and interventions.

### Recommendations

To improve anaemia and malnutrition in children less than 5 years of age in Lesotho, policy makers, donors and government should focus on improving nutrition status especially, in children from rural area, with diarrhoea, fever, low birthweight, from poorer quantile index household and uneducated mother.

## Data Availability

The present study utilized existing dataset and is available from https://www.dhsprogram.com/data/dataset_admin/login_main.cfm with the permission from the DHS program.

## Abbreviations

OR: Odds ratio
SE: Standard error
95% CI: Confidence Interval
LDHS: Lesotho demographic health survey
GLMM: Generalized linear mixed model
MVN: Multivariate normal distribution
AIC: Akaike information criterion
Hb: Haemoglobin concentration
UN: Unstructured
WHO: World Health Organization
SD: Standard deviations
HAZ: Height-for-age z score, SAM: severe acute malnutrition
MAM: Moderate acute malnutrition
UNICEF: United nations international children's emergency fund
MOHSW: Ministry of health and family welfare
HIV: Human immunodeficiency virus
AIDS: Acquired immunodeficiency syndrome
EAs: Enumeration areas

## References

[1] Adebayo SB, Gayawan E, Heumann C, Seiler C (2016). Joint modeling of Anaemia and Malaria in children under five in Nigeria. Spatial and spatio-temporal epidemiology.

[2] Adeyemi RA, Zewotir T, Ramroop S (2019). Joint spatial mapping of childhood anaemia and malnutrition in sub-Saharan Africa: a cross-sectional study of small-scale geographical disparities. Afr Health Sci.

[3] Adhikari RP, Shrestha ML, Acharya A, Upadhaya N (2019). Determinants of stunting among children aged 0–59 months in Nepal: findings from Nepal Demographic and Health Survey, 2006, 2011, and 2016. BMC nutrition.

[4] Agresti A (2015). Foundations of linear and generalized linear models.

[5] Aheto JMK, Keegan TJ, Taylor BM, Diggle PJ (2015). Childhood Malnutrition and Its Determinants among Under-Five Children in Ghana. Paediatr Perinat Epidemiol.

[6] Aheto JMK, Taylor BM, Keegan TJ, Diggle PJ (2017). Modelling and forecasting spatio-temporal variation in the risk of chronic malnutrition among under-five children in Ghana. Spatial and spatio-temporal epidemiology.

[7] Anticona C, San Sebastian M (2014). Anemia and malnutrition in indigenous children and adolescents of the Peruvian Amazon in a context of lead exposure: a cross-sectional study. Glob Health Action.

[8] Bardosono S, Sastroamidjojo S, Lukito W. Determinants of child malnutrition during the 1999 economic crisis in selected poor areas of Indonesia. Asia Pacific journal of clinical nutrition. 2007;16(3):512-26. 17704034

[9] Bechir M, Schelling E, Hamit MA, Tanner M, Zinsstag J (2012). Parasitic infections, anaemia and malnutrition among rural settled and mobile pastoralist mothers and their children in Chad. EcoHealth.

[10] Black RE, Victora CG, Walker SP, Bhutta ZA, Christian P, De Onis M, Ezzati M, Grantham-McGregor S, Katz J, Martorell R, Uauy R (2013). Maternal and child undernutrition and overweight in low-income and middle-income countries. The lancet.

[11] Breslow NE, Clayton DG (1993). Approximate inference in generalized linear mixed models. J Am Stat Assoc.

[12] El Kishawi RR, Soo KL, Abed YA, Muda WAMW (2015). Anaemia among children aged 2–5 years in the Gaza Strip-Palestinian: a cross sectional study. BMC Public Health.

[13] Gari T, Loha E, Deressa W, Solomon T, Atsbeha H, Assegid M, Hailu A, Lindtjørn B (2017). Anaemia among children in a drought affected community in south-central Ethiopia. PloS one.

[14] Gaston RT, Ramroop S, Habyarimana F. Determinants of factors associated with anaemia among children under five years in Lesotho. African Population Studies. 2018;32(1):3893-902.

[15] Gaston RT, Ramroop S (2020). Prevalence of and factors associated with malaria in children under five years of age in Malawi, using malaria indicator survey data. Heliyon.

[16] Gaston RT, Ramroop S, Habyarimana F (2021). Joint modelling of malaria and anaemia in children less than five years of age in Malawi. Heliyon.

[17] Gayawan E, Arogundade ED, Adebayo SB (2014). Possible determinants and spatial patterns of anaemia among young children in Nigeria: a Bayesian semi-parametric modelling. Int Health.

[18] Gueorguieva R (2001). A multivariate generalized linear mixed model for joint modelling of clustered outcomes in the exponential family. Stat Model.

[19] Habyarimana F, Zewotir T, Ramroop S (2016). Joint modeling of poverty of households and malnutrition of children under five years from demographic and health survey data: case of Rwanda. Journal of Economics and Behavioral Studies.

[20] Hedeker D. Generalized linear mixed models. In: Everitt, B., Howell, D. (Eds.), Encyclopedia of statistics in behavioral science. New York: Wiley; 2005. p. 729-738.

[21] Kassebaum N, Kyu HH, Zoeckler L, Olsen HE, Thomas K, Pinho C, Bhutta ZA, Dandona L, Ferrari A, Ghiwot TT, Hay SI (2017). Child and adolescent health from 1990 to 2015: findings from the global burden of diseases, injuries, and risk factors 2015 study. JAMA Paediatrics.

[22] Kavosi E, Rostami ZH, Kavosi Z, Nasihatkon A, Moghadami M, Heidari M (2014). Prevalence and determinants of under-nutrition among children under six: a cross-sectional survey in Fars province. Iran International Journal of Health Policy and Management.

[23] Kazembe LN (2013). An additive regression model for investigating the relationship between childhood health and socio-economic status. Spatial and Spatio-temporal Epidemiology.

[24] Kejo D, Petrucka PM, Martin H, Kimanya ME, Mosha TC (2018). Prevalence and predictors of anemia among children under 5 years of age in Arusha District, Tanzania. Pediatric health, medicine and therapeutics.

[25] Kounnavong S, Sunahara T, Mascie-Taylor CN, Hashizume M, Okumura J, Moji K, Boupha B, Yamamoto T (2011). Effect of daily versus weekly home fortification with multiple micronutrient powder on haemoglobin concentration of young children in a rural area, Lao People's Democratic Republic: a randomised trial. Nutr J.

[26] Leal LP, Batista Filho M, Lira PICD, Figueiroa JN, Osório MM (2011). Prevalence of anaemia and associated factors in children aged 6–59 months in Pernambuco, Northeastern Brazil. Rev Saude Publica.

[27] Letsie MM, Grab SW. Assessment of social vulnerability to natural hazards in the Mountain Kingdom of Lesotho. Mountain Research and Development. 2015;5(2):115-25.

[28] Letuka T, Frade S (2020). Household and individual risk factors of anaemia among under-5 children in Lesotho. Afr Health Sci.

[29] Masibo PK, Makoka D (2012). Trends and determinants of undernutrition among young Kenyan children: Kenya Demographic and Health Survey; 1993, 1998, 2003 and 2008–2009. Public Health Nutr.

[30] Milman N (2011). Anaemia-still a major health problem in many parts of the world!. Ann Hematol.

[31] Ministry of Health [Lesotho] and ICF International (2016). Lesotho Demographic and Health Survey 2014.

[32] Mohammed SH, Larijani B, Esmaillzadeh A (2019). Concurrent anemia and stunting in young children: prevalence, dietary and non-dietary associated factors. Nutr J.

[33] Molenberghs G, Verbeke G (2005). Models for discrete longitudinal data.

[34] Okoromah CA, Ekure EN, Lesi FE, Okunowo WO, Tijani BO, Okeiyi JC (2011). Prevalence, profile and predictors of malnutrition in children with congenital heart defects: a case–control observational study. Arch Dis Child.

[35] Oliveira D, Ferreira FS, Atouguia J, Fortes F, Guerra A, Centeno-Lima S (2015). Infection by intestinal parasites, stunting and anemia in school-aged children from southern Angola. PloS one.

[36] Olney DK, Kariger PK, Stoltzfus RJ, Khalfan SS, Ali NS, Tielsch JM, Sazawal S, Black R, Allen LH, Pollitt E (2009). Development of nutritionally at-risk young children is predicted by malaria, anaemia, and stunting in Pemba. Zanzibar The Journal of Nutrition.

[37] Rahman MS, Mushfiquee M, Masud MS, Howlader T (2019). Association between malnutrition and anaemia in under-five children and women of reproductive age: Evidence from Bangladesh Demographic and Health Survey 2011. PloS one.

[38] Renzaho AMN. Mortality, malnutrition, and the humanitarian response to the food crises in Lesotho. Journal of Emergency Primary Health Care. 2006;4(4):1-15.

[39] Rivadeneira MF, Moncayo AL, Tello B, Torres AL, Buitrón GJ, Astudillo F, Fredricks TR, Grijalva MJ (2020). A Multi-Causal Model for Chronic Malnutrition and Anaemia in a Population of Rural Coastal Children in Ecuador. Matern Child Health J.

[40] Sachdev HPS, Gera T, Nestel P (2005). Effect of iron supplementation on mental and motor development in children: systematic review of randomised controlled trials. Public Health Nutr.

[41] Tuerlinckx F, Rijmen F, Verbeke G, De Boeck P (2006). Statistical inference in generalized linear mixed models: A review. Br J Math Stat Psychol.

[42] UNICEF (2017). State of the World’s children: Children in a digital World.

[43] Varghese JS, Stein AD (2019). Malnutrition among women and children in India: limited evidence of clustering of underweight, anemia, overweight, and stunting within individuals and households at both state and district levels. Am J Clin Nutr.

[44] Verbeke G, Molenberghs G (2003). The use of score tests for inference on variance components. Biometrics.

[45] Worku Z (2003). Malnutrition among rural and urban children in Lesotho: related hazard and survival probabilities. Health SA Gesondheid.

[46] World Health Organization (2007). WHO child growth standards: head circumference-for-age, arm circumference-for-age, triceps skinfold-for-age and subscapular skinfold-for-age: methods and development.

[47] World Health Organization (2011). Haemoglobin concentrations for the diagnosis of anaemia and assessment of severity.

[48] Yang W, Li X, Li Y, Zhang S, Liu L, Wang X, Li W (2012). Anemia, malnutrition and their correlations with socio-demographic characteristics and feeding practices among infants aged 0–18 months in rural areas of Shaanxi province in north western China: a cross-sectional study. BMC Public Health.

